# New Model for Estimating Glomerular Filtration Rate in Patients With Cancer

**DOI:** 10.1200/JCO.2017.72.7578

**Published:** 2017-07-07

**Authors:** Tobias Janowitz, Edward H. Williams, Andrea Marshall, Nicola Ainsworth, Peter B. Thomas, Stephen J. Sammut, Scott Shepherd, Jeff White, Patrick B. Mark, Andy G. Lynch, Duncan I. Jodrell, Simon Tavaré, Helena Earl

**Affiliations:** Tobias Janowitz, Edward H. Williams, Stephen J. Sammut, Andy G. Lynch, Duncan I. Jodrell, Simon Tavaré, and Helena Earl, Cancer Research UK Cambridge Institute, Tobias Janowitz, Peter B. Thomas, and Duncan I. Jodrell, University of Cambridge, Addenbrooke’s Hospital, Cambridge; Andrea Marshall, University of Warwick, Coventry; Nicola Ainsworth, Queen Elizabeth Hospital, King’s Lynn; Scott Shepherd, Royal Marsden Hospital, London; Jeff White, NHS Greater Glasgow and Clyde; and Patrick B. Mark, Institute of Cardiovascular and Medical Sciences, University of Glasgow, Glasgow, United Kingdom.

## Abstract

**Purpose:**

The glomerular filtration rate (GFR) is essential for carboplatin chemotherapy dosing; however, the best method to estimate GFR in patients with cancer is unknown. We identify the most accurate and least biased method.

**Methods:**

We obtained data on age, sex, height, weight, serum creatinine concentrations, and results for GFR from chromium-51 (^51^Cr) EDTA excretion measurements (^51^Cr-EDTA GFR) from white patients ≥ 18 years of age with histologically confirmed cancer diagnoses at the Cambridge University Hospital NHS Trust, United Kingdom. We developed a new multivariable linear model for GFR using statistical regression analysis. ^51^Cr-EDTA GFR was compared with the estimated GFR (eGFR) from seven published models and our new model, using the statistics root-mean-squared-error (RMSE) and median residual and on an internal and external validation data set. We performed a comparison of carboplatin dosing accuracy on the basis of an absolute percentage error > 20%.

**Results:**

Between August 2006 and January 2013, data from 2,471 patients were obtained. The new model improved the eGFR accuracy (RMSE, 15.00 mL/min; 95% CI, 14.12 to 16.00 mL/min) compared with all published models. Body surface area (BSA)–adjusted chronic kidney disease epidemiology (CKD-EPI) was the most accurate published model for eGFR (RMSE, 16.30 mL/min; 95% CI, 15.34 to 17.38 mL/min) for the internal validation set. Importantly, the new model reduced the fraction of patients with a carboplatin dose absolute percentage error > 20% to 14.17% in contrast to 18.62% for the BSA-adjusted CKD-EPI and 25.51% for the Cockcroft-Gault formula. The results were externally validated.

**Conclusion:**

In a large data set from patients with cancer, BSA-adjusted CKD-EPI is the most accurate published model to predict GFR. The new model improves this estimation and may present a new standard of care.

## INTRODUCTION

The glomerular filtration rate (GFR), the fluid volume filtered from the capillaries of the renal glomeruli into the Bowman’s capsule per unit time, is used for calculations of carboplatin chemotherapy doses.^1^ A number of direct GFR measurements exist, such as the calculation on the basis of clearance of chromium-51 EDTA (^51^Cr-EDTA).^2^ These methods are costly and require time and expertise. As a substitute, models for GFR estimation have been developed on the basis of readily available data, such as serum creatinine concentrations, age, and sex of the patient.^3-11^

These published models for GFR have been mainly developed for noncancer patient populations that are frequently enriched for patients with chronic kidney disease. Their usefulness in patients with cancer has been examined using only small data sets, and limitations have been documented.^12-16^

Uncertainties regarding GFR estimation for patients with cancer represent an area of clinical need. Carboplatin chemotherapy doses calculated using GFR^1^ are administered to patients with seminoma, lung, breast, and ovarian cancer, in both adjuvant and palliative settings, where accurate dosing is critical to both outcome and toxicity.^17-27^ In addition, GFR measurements guide clinicians with regard to cisplatin use, which is nephrotoxic^28,29^ and considered with caution in patients with reduced renal function.^30-32^ We used the largest published oncology data set to identify the most accurate published model as well as to develop a new model to estimate GFR.

## METHODS

Detailed methods and a comprehensive description of development of the new model are provided in the Data Supplement.

### Study Profile and Data Set

**Fig 1.**
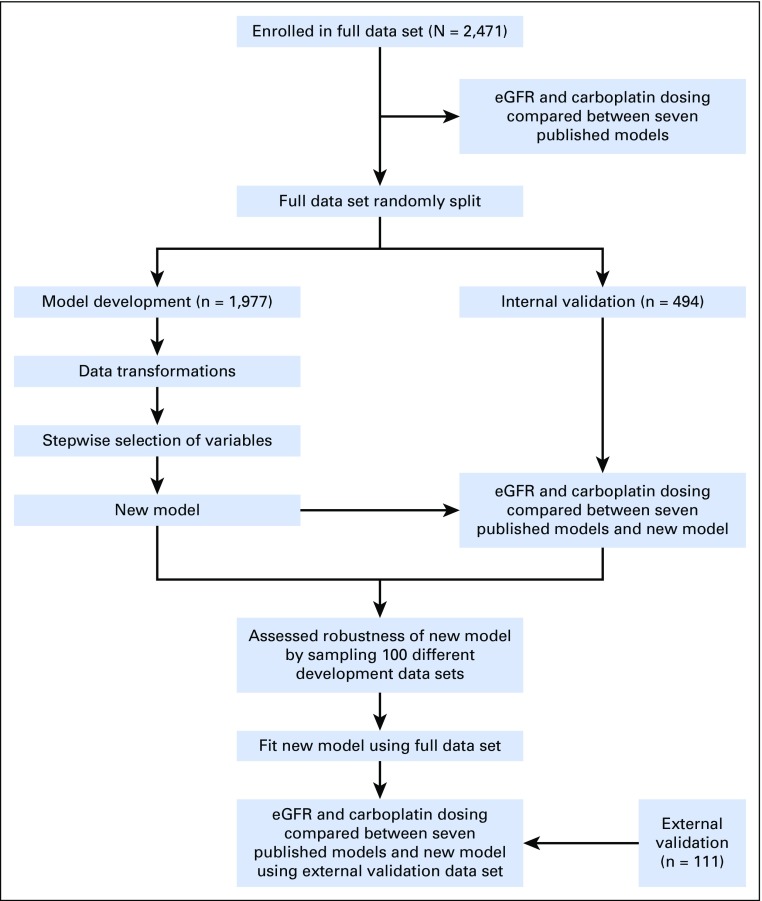
Schematic representation of study workflow. eGFR, estimated glomerular filtration rate.

The study profile is displayed schematically in Figure 1. The full data set was compiled at the Cambridge University Hospital NHS Trust, United Kingdom, from white patients ≥ 18 years of age with histologically confirmed cancer diagnoses and a serum creatinine measurement within 30 days of the ^51^Cr-EDTA GFR–measurement (^51^Cr-EDTA GFR). The data set was randomly split at a ratio of 4:1 for model development and internal model validation. An external validation data set of male patients (n = 111) with stage I seminoma was obtained from the Beatson West of Scotland Cancer Centre, Glasgow, United Kingdom. No patient-identifiable data were used. Anonymized data included age, sex, height, weight, serum creatinine concentration, and results for the accurate GFR value from ^51^Cr-EDTA GFR. Body surface area (BSA) was calculated using the Du Bois equation.^33^ Height, weight, and ^51^Cr-EDTA GFR were measured on the same day.

### Assessment of Published Models

We compared the ^51^Cr-EDTA GFR with the GFR estimated using the following five published models, with and without BSA adjustment: Martin, Wright, Mayo, Modification of Diet in Renal Disease (MDRD), and chronic kidney disease epidemiology (CKD-EPI). The Cockcroft-Gault and the Jelliffe models, which estimate creatinine clearance (ie, an approximation of GFR), were also assessed.^3-10^

We used the Calvert equation^1^ to compare the accuracy of a carboplatin dose with an area under the curve (AUC) of 5 mg/mL/min (AUC5) calculated from ^51^Cr-EDTA GFR with eGFR for all models.

### Model Generation

In brief, we developed a linear model for the relationship between GFR and the predicting variables. The Box-Cox method^34^ gave a suitable transformation to approximate normality. The model variables were chosen using minimization of a five-fold cross validation, a leave-one-out cross validation, and the Bayesian information criterion in a stepwise method starting from a model containing only an intercept term (null model).^35-40^ To address the random component associated with this selection process for the five-fold cross-validation criterion, 2,000 repetitions of the process were performed and the most frequent model was taken forward.

### Laboratory Methods and GFR Calculation

GFR was calculated from the measurement of ^51^Cr-EDTA in three plasma samples taken over time after intravenous injection of 2 megabecquerel (MBq) of ^51^Cr-EDTA. Serum creatinine (Cre) was measured using the kinetic Jaffe method.

### Statistics

Median percentage error (PE), root-mean-squared error (RMSE), interquartile range (IQR) of the residuals, and median absolute percentage error (APE) were used to assess the accuracy of each GFR model for predicting measured ^51^Cr-EDTA GFR. A median APE > 20% was considered a clinically relevant deviation of the carboplatin dose. RMSE results are expressed with a 95% CI calculated using the χ^2^ distribution. All median statistics are reported with IQRs.

## RESULTS

Between August 2006 and January 2013, data from 2,471 patients were obtained. The data set was divided randomly into data from 1,977 patients (80%) for model development and from 494 patients (20%) for internal validation of the new model. The patient characteristics were similar between the different data sets and are summarized in Table 1. Serum creatinine and ^51^Cr-EDTA GFR were measured within 30 days (median, 6 days; IQR, 2 to 9 days). The median for ^51^Cr-EDTA GFR was 81 mL/min (IQR, 63 to 103 mL/min), indicating that most patients had near-normal kidney function.^41^ The external validation data set consisted of patients with stage I seminoma (n = 111), who had a median age of 39 years (IQR, 33 to 46 years) and a median ^51^Cr-EDTA GFR of 113 mL/min (IQR, 101 to 131 mL/min; Table 1).

**Table 1.**
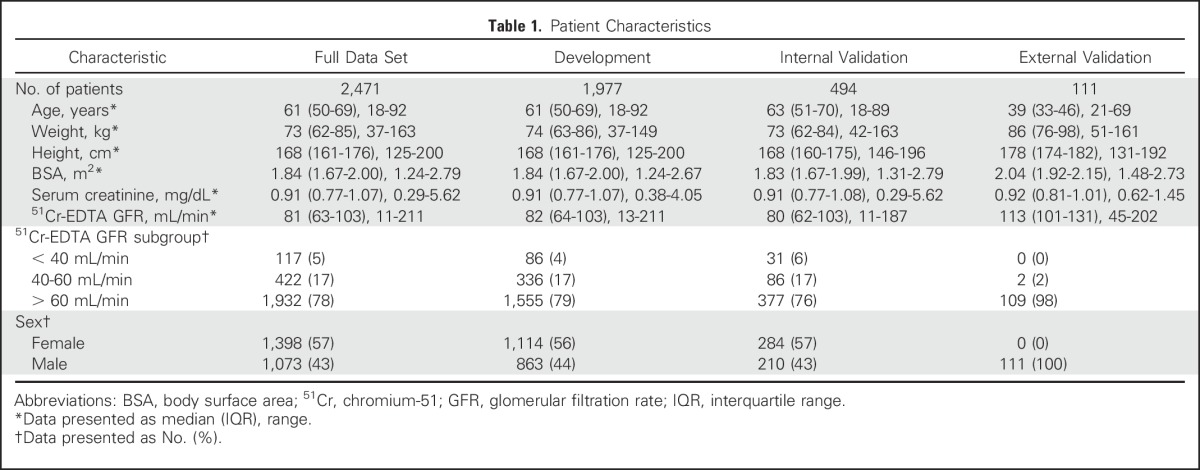
Patient Characteristics

We used the full data set to compare the performance of seven published candidate models and BSA-adjusted models (Mayo, Jelliffe, MDRD, and CKD-EPI). For estimating GFR, CKD-EPI was the most accurate model, with the lowest RMSE at 21.17 mL/min (95% CI, 20.60 to 21.78 mL/min). BSA adjustment improved accuracy for the CKD-EPI, MDRD, and Jelliffe models. After BSA adjustment, CKD-EPI had the lowest RMSE (16.63 mL/min; 95% CI, 16.18 to 17.10 mL/min), was least biased (median residual, 0.54 mL/min; IQR, −10.18 to 9.16 mL/min), and had a median PE closest to zero (−0.78%; IQR, −14.09% to 11.19%), the smallest residual IQR (19.34 mL/min), and the smallest median APE (12.33%; IQR, 5.77% to 21.62%).

With regard to carboplatin doses, calculated by the Calvert equation: dose [mg] = Target AUC [mg/mL/min] × (GFR [mL/min] + 25 [mL/min]),^1^ where dose is linearly related to GFR, the statistics of RMSE, median residual, and IQR of residuals are direct reflections of the GFR results but median PE and median APE are different. We determined the fraction of patients receiving doses with a clinically relevant APE > 20%, which was smallest for BSA-adjusted CKD-EPI (17.38%). BSA-adjusted CKD-EPI, therefore, was the best-performing published model for estimation of GFR and calculation of carboplatin dose in our data set from patients with cancer (Data Supplement).

Next, we investigated if our large data set could be used to develop a new and better model. We first noticed that the untransformed GFR data were not normally distributed (Data Supplement). The Box-Cox method suggested that modeling the square root of GFR would satisfy the assumptions of a linear model (Data Supplement). The relationship between square root GFR and untransformed creatinine was not linear (Data Supplement). Of several tested data transformations, natural logarithmic transformation (ln) achieved the best linearity between GFR and the transformed creatinine (Data Supplement). However, graphical analysis of the residual against transformed serum creatinine concentration for a simple model (ie, a model that had the variables ln(Cre), sex, and BSA) showed that further transformations were required (Data Supplement). Including a quadratic and cubic term further improved the linearity, better modeled the complex relationship (Data Supplement), and significantly improved the model (*P* < .001, F-test). Age, BSA, height, and weight had an approximately linear relationship with square root GFR (Data Supplement).

For model selection on the development data set, we used the leave-one-out, five-fold, and Bayesian information criteria. All three of our criteria selected the same model (Eq 1). The five-fold criterion selected the model 854 times out of the 2,000 repetitions.

**Fig 2.**
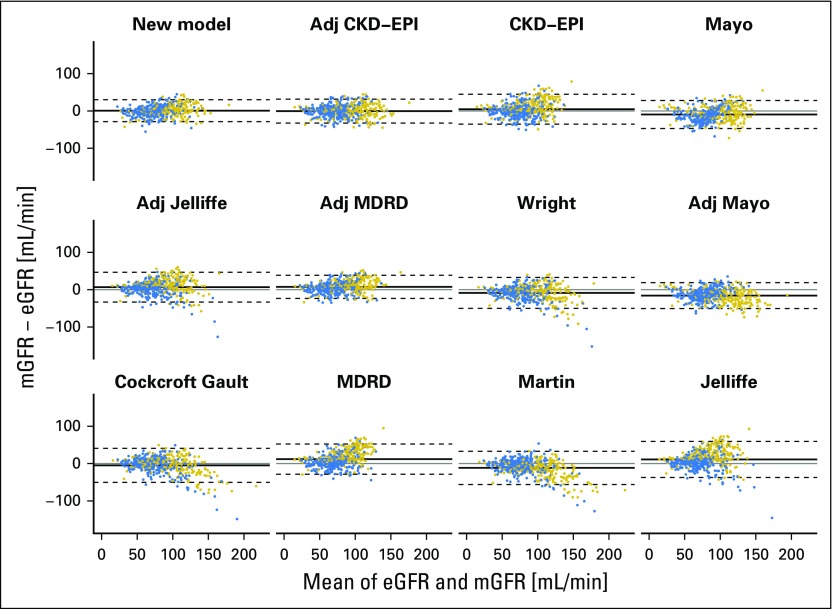
Bland-Altman plots of estimated GFR (eGFR) and measured GFR (mGFR) for the new model and each of the published models. The mean of mGFR and eGFR was plotted against the difference of the two for the internal validation data set. Positive differences indicate underestimation and negative differences indicate overestimation. The plots are ordered in ascending order of root-mean-squared error of eGFR from top left to bottom right. The solid line on each plot represents the mean of the difference and the dashed lines are drawn at the mean ± 1.96 times the standard deviation of the difference. Points are colored by sex (blue, female; gold, male). Adj, adjusted; CKD-EPI, chronic kidney disease epidemiology; MDRD, Modification of Diet in Renal Disease.

**Fig 3.**
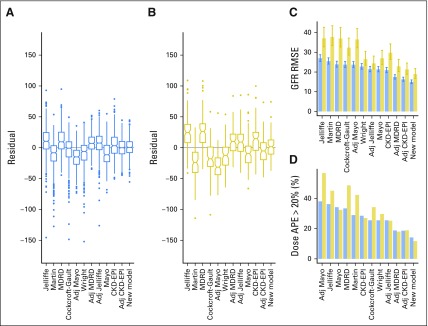
Graphical illustrations of statistics used to compare the new model and published models. Box plots of the residuals (measured glomerular filtration rate [GFR] minus estimated GFR) for all published models and the new model using (A) the internal validation data set and (B) the external validation data set are shown. Notches delineate an approximate 95% CI for the median residual, calculated as ± 1.58*interquartile range/n^0.5^. A positive or negative value for the median residual indicates underestimation or overestimation bias, respectively. (C) Graphical illustration of GFR root-mean-squared error (RMSE) in the internal and external validation data sets. Error bars describe the 95% CI on the basis of the χ^2^ distribution for the calculated RMSE. (D) Graphical illustration of percentage of patients with a carboplatin dosing absolute percentage error (APE) > 20% in the internal and external validation data sets. For all plots, blue represents the internal validation set and gold represents the external validation data set. Adj, adjusted; CKD-EPI, chronic kidney disease epidemiology; MDRD, Modification of Diet in Renal Disease.

Using the internal validation data set, we compared the performance of the new model with the performance of the published models. Bland-Altman and residual plots indicated that the new model is more accurate, less biased, and less heteroscedastic, ie, it has more constant variance in different subpopulations (Fig 2 and Data Supplement). These plots also demonstrate that the new model, CKD-EPI, and BSA-adjusted CKD-EPI are least prone to overfitting. The new model was the most accurate and second least biased model for estimating GFR (Fig 3A and C, Data Supplement). It has the lowest RMSE at 15.00 mL/min (95% CI, 14.12 to 16.00 mL/min) and a median residual of 0.51 mL/min (IQR, −7.99 to 9.67 mL/min). For the BSA-adjusted CKD-EPI model, the RMSE and the median residual were 16.30 mL/min (95% CI, 15.34 to 17.38 mL/min) and −0.03 mL/min (IQR, −9.92 to 10.13 mL/min), respectively, and for the Cockcroft-Gault model, the RMSE and the median residual were 23.75 mL/min (95% CI, 22.36 to 25.33 mL/min) and −0.79 mL/min (IQR, −14.93 to 9.54 mL/min), respectively (Fig 3C).

We consider use of the new model for calculation of carboplatin dosing for patients with cancer to be the most important area of potential clinical application. Thus we investigated the fraction of patients who would have received an AUC5 carboplatin dose that deviated > 20% from the accurate dose calculated using ^51^Cr-EDTA GFR. This fraction was smallest for the new model, with an APE > 20% of 14.17% in contrast to 18.62% for the BSA-adjusted CKD-EPI and 25.51% for the Cockcroft-Gault model (Fig 3D and Data Supplement).

We also investigated utility of the new model to guide prescription of cisplatin, which is an important chemotherapeutic agent but causes nephrotoxicity.^28,29^ Of the 58 patients within the internal validation data set who had a measured GFR < 50 mL/min (a value that warrants caution for full-dose cisplatin administration),^30-32^ the new model returned an eGFR lower than this value for 31 (53%) patients. This compares with 36 (62%) and 35 (60%) of patients when the BSA-adjusted CKD-EPI or the Cockcroft-Gault model was used, respectively. In turn, of the 436 patients who had a measured GFR > 50 mL/min, a total of nine patients (2.1%, new model), 16 patients (3.7%, BSA-adjusted CKD-EPI model), and 29 patients (6.7%, Cockcroft-Gault model) had an eGFR < 50 mL/min.

**Fig 4.**
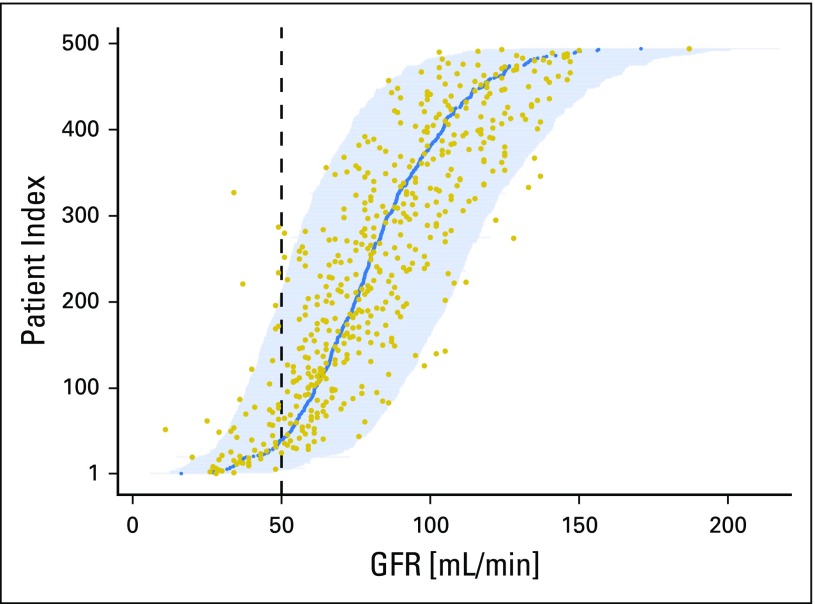
Predictive CIs for glomerular filtration rate (GFR) of each patient in the internal validation data set. To obtain this figure, the new model fitted on the development data set was applied to all patients in the internal validation data set. The measured GFR (gold points) and the estimated GFR (blue points) for each patient are illustrated. Each horizontal line represents a 95% predictive CI for the patient, with patients ordered in accession by their estimated GFR. The vertical dashed line highlights the boundary at a GFR of 50 mL/min, below which cisplatin administration would be considered with caution by most clinicians. Of the 494 patients in the internal validation data set, 24 (4.9%) had measured values outside their prediction interval.

This demonstrates limitations of point estimates. However, the new model satisfies all linear modeling assumptions; therefore, predictive CIs for future unobserved GFR values can be estimated (Fig 4). For 54 (93%) of the 58 patients with a measured GFR < 50 mL/min, the 95% predictive CI includes 50 mL/min. This increased detection rate of at-risk patients is offset by predictive CIs that contain 50 mL/min for 158 (36%) patients with a measured GFR > 50 mL/min.

To assess robustness of the model, the variable selection process was repeated for the three criteria on 100 different random partitions of the full data set into development and validation data sets. The new model remained most frequently returned and has the form

Equation 1: 

$$\begin{array}{l} \sqrt{\text{GFR}}=\beta_{0}+\beta_{1}\text{Age}+\beta_{2}\text{BSA}+\beta_{3}\text{ln}\left(\text{Cre}\right)+\beta_{4}\text{ln}{\left(\text{Cre}\right)}^{2}+\beta_{5}\text{ln}{\left(\text{Cre}\right)}^{3}\\ +\left(\beta_{6}+\beta_{7}\text{Age}\right)\left\{\text{if Sex}=M\right\}+\beta_{8}\text{Age}\times \text{BSA}+\varepsilon \end{array}$$

where the errors *ε* are independent, mean zero normally distributed random variables with a constant variance *σ*^2^. To get the most accurate values, the final coefficients *β*_0_, …, *β*_8_ were determined by fitting the model using the full data set (Table 2).

**Table 2.**
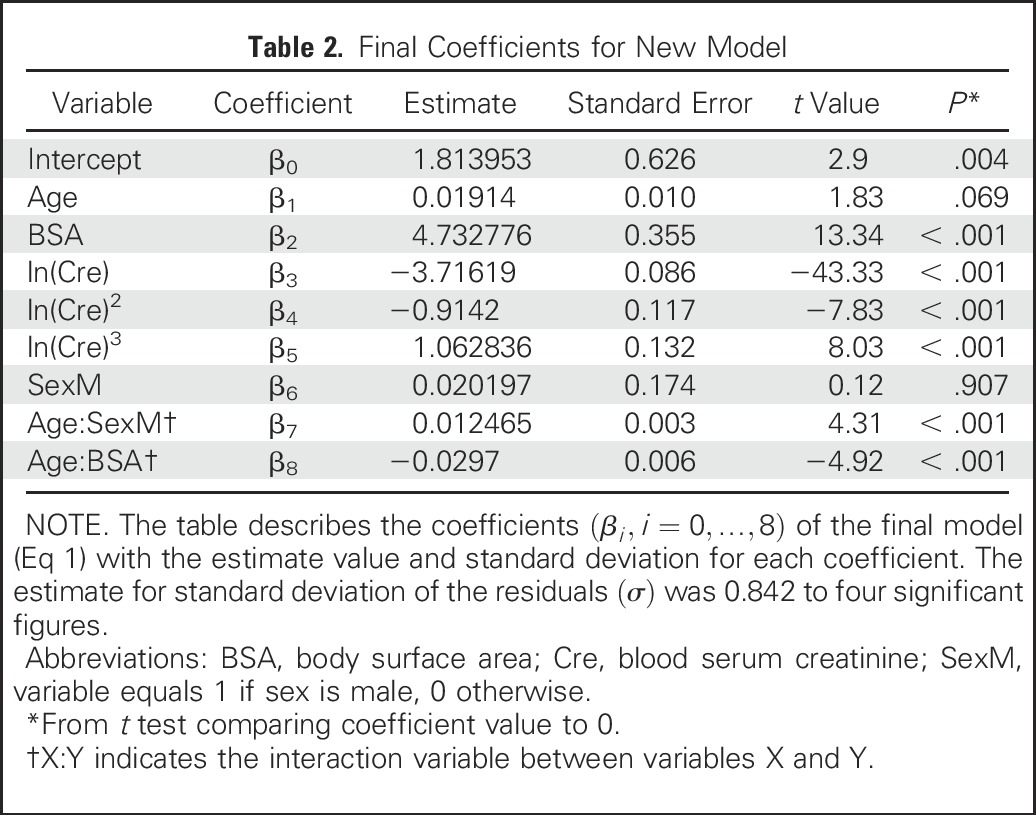
Final Coefficients for New Model

Diagnostic plots for the new model confirmed that no single data point was influential in the full data set (highest Cook’s distance value, 0.094; Data Supplement). Importantly, there was still no heteroscedasticity in the final linear model; thus we confirmed that calculation of CIs (prediction intervals) for the eGFR values were appropriate (Data Supplement).

Finally, we externally validated the model using a data set from a different cancer center. GFR estimation (Data Supplement) and dose accuracy assessment for carboplatin demonstrated that the new model remained the most accurate compared with all other models. The RMSE for the GFR calculated with the new model was 18.94 mL/min compared with 21.33 mL/min for the BSA-adjusted CKD-EPI and 32.32 mL/min for the Cockcroft-Gault model (Fig 3B and C and Data Supplement). The carboplatin AUC5 APE > 20% was 11.71% for the new model and 18.92% for the BSA-adjusted CKD-EPI model, which was the next best model (Fig 3D and Data Supplement). Of the 111 patients in the external validation data set, 105 (94.6%) had a measured GFR within the 95% CI (Data Supplement).

## DISCUSSION

Our work is based on analysis of data from a total of 2,582 patients with cancer and reports two potentially practice-changing results. First, we found that the BSA-adjusted CKD-EPI was the most accurate and least biased published model to estimate GFR. Second, we developed a new model that further improves the estimation of GFR and allows calculation of predictive CIs for this estimation. Both findings will help practicing oncologists who prescribe platinum-based chemotherapy.

Determination of GFR is a cornerstone of the curative and palliative management of patients with carboplatin-responsive cancer such as lung, ovary, triple-negative– and germline BRCA1/2 mutation–positive breast cancer, and seminomas.^18-27^ Carboplatin doses are most commonly calculated using the Calvert equation,^1^ which is a linear relationship between GFR and dose. GFR measurements or estimates therefore directly influence dose accuracy. This is important because carboplatin is dose-dependently linked to tumor response and toxicities.^17^ Methods to measure GFR after tracer injection^2^ are laborious, expensive, and not considered routine clinical investigations. Consequently, oncologists often rely on methods to estimate GFR from biometric patient data and routine blood test results, most notably serum creatinine concentration. With the exception of the Wright equation,^8^ which investigated 100 patients with cancer, these methods have been developed for purposes other than chemotherapy dosing and with data from noncancer populations, which are enriched for patients with impaired kidney function compared with our data set.

Practice-changing clinical trials of carboplatin chemotherapy have used gold standard ^51^Cr-EDTA GFR measurements,^18-20^ creatinine clearance using the Cockcroft-Gault model,^21-23^ the Jelliffe model,^24-27,42^ or 24-hour urine creatinine collections.^19^ This demonstrates absence of a consensus. The findings of our study show that of the published methods, unadjusted CKD-EPI predicts GFR (and consequently carboplatin doses) similarly well to the Jelliffe, Wright, and Cockcroft-Gault models in patients with cancer. We confirmed the finding of other studies that the inclusion of BSA in predictive models improves accuracy.^12^ BSA-adjusted CKD-EPI had the lowest RMSE and bias, as well as the smallest carboplatin dose APE > 20%, and should, therefore, be considered the best published creatinine-based GFR estimation model.

Patients in the development group for the CKD-EPI model^10^ were noncancer patients and had a mean GFR of 68 mL/min/1.73 m^2^; thus they were different than the patient population in our study (ie, the population of patients with cancer who were scheduled to receive carboplatin or cisplatin chemotherapy). We hypothesized that we could derive a new model to better predict GFR for patients with cancer. We recognized that there are multiple approaches to developing a model for the relationships among the dependent variable, GFR, and the independent predicting variables. Square root–transformed GFR is an approximately normally distributed variable, its relationship to the independent variables is approximately linear, and the resulting residuals have a mean of zero and constant variance. Therefore, we concluded that a linear model with square-root transformation of GFR was appropriate. Evidence from our internal and external validation work suggests that our new model is the best currently available model to predict GFR in patients with cancer.

From a clinical point of view, the most important advantage of our new model is a reduction in the fraction of patients who receive a carboplatin dose that is > 20% different from the dose calculated using ^51^Cr-EDTA GFR, even when compared with the BSA-adjusted CKD-EPI model. The absolute reduction in the external validation set ranged from 34.23% for the Cockcroft-Gault and 18.92% for the BSA-adjusted CKD-EPI model to 11.71% with the new model. In addition, we report the mean of the prediction as well as the variance and, therefore, the predictive CI. This represents a further advantage because it will provide clinicians with a gauge of the suitability of using the prediction in a given clinical context. For example, our analysis demonstrated that only four of 58 patients from the validation data set with a measured GFR < 50 mL/min did not contain this value in the 95% predictive CI. We present the data for the value 50 mL/min, but recognize that this value would be dependent on the clinical context.^30-32^ We acknowledge this fact by providing an estimated probability of the patient’s true GFR being below a user-adjustable GFR value as part of an online tool to offer a clinical guide for prescription of cisplatin, which is a nephrotoxic chemotherapeutic.^28,29^

Further strengths of our study are the large data set from patients with cancer, the stringent methodology, and the internal and external validation of our findings. Our model is based on standard biometric data and can be easily used in clinical practice. The study is limited, however, by the white-only population, as a result of the single-center population demographics. Others have shown that adjustment factors improve GFR prediction for black patients,^6,10^ and this should be a priority area for future investigations. The final coefficients reported in our study may be, to a degree, center dependent as a result of center-dependent creatinine results. This is a problem that has been addressed by international guidelines to standardize creatinine reporting,^43^ which are implemented at our center. Using creatinine as the main explanatory variable in predicting GFR has its own limitations. Other predicting variables (eg, cystatin C) have been used, but were not available to us. Furthermore, their usefulness in patients with cancer is uncertain, because their levels may fluctuate in a cancer-dependent and kidney function–independent manner.^44^ We also did not analyze measurements of albumin, muscle mass, information on dietary and fluid intake, and comorbidities such as diabetes mellitus.

Our findings may be relevant for a broad range of clinical decision making in patients with or without cancer diagnoses. GFR influences clinical management in the context of drug dose adjustments^45^ and decision making in the context of clinical organ support.^41^ Gold standard ^51^Cr-EDTA GFR measurement would usually not be performed in these contexts. Future research should also investigate if the new model can facilitate correlative and ultimately causative analysis of toxicity and dose accuracy relationships in clinical trials.

In conclusion, BSA-adjusted CKD-EPI is the most accurate published model to estimate GFR in patients with cancer. Our new model further improves the estimation accuracy for GFR and may present a new standard of care and should be investigated alongside BSA-adjusted CKD-EPI in clinical practice.

## ONLINE TOOL

The new model has been implemented in an online tool.^46^ For any given set of input data, the tool provides the eGFR according to the new model, an estimated predictive CI for the true GFR (default setting at 95%), an estimated probability of the true GFR being below or above an operator-chosen value (default setting at 50 mL/min), as well as the eGFR according to the BSA-adjusted CKD-EPI model.
